# Individualized composition or community dynamics? A new statistical approach to assess the individuality of host-associated microbiomes

**DOI:** 10.1098/rspb.2022.1794

**Published:** 2022-11-09

**Authors:** Elizabeth K. Mallott

**Affiliations:** Department of Biology, Washington University in St Louis, St Louis, MO 63130, USA

Studies of the gut microbiome across mammals highlight both individualized microbial signatures and highly dynamic microbial communities. While it is certainly possible that a host-associated microbial community could be both dynamic over time but also individualized, it may be more likely that highly dynamic microbial communities become less individualized over time. This question of when and why microbiomes are individualized is a key to understanding how eco-evolutionary processes shape microbial community composition.

The individuality of the microbiome has been examined previously over various timescales, but primarily using comparisons of within versus between individual similarity. Some of these studies show a large effect of individual identity on the microbiome over a period of two months [1], 1.5 years [2], 2 years [3] or 8 years [4]. However, in two of these cases [2,4], the effects were time dependent. Other longitudinal studies of the microbiome where individuals were repeatedly sampled over long periods of time include individual identity as a random effect in their statistical models to control for individual variation, but do not directly test what proportion of the variation is explained by identity [5–7]. While some studies in the human literature emphasize a repeatable, individualized gut microbial signature [3], more recent work supports an individualized gut microbiome over shorter timescales where strain evolution is a primary driver of community composition, but that strain replacement dominates over multi-decade timescales [8].

Risely *et al.* [9] tackle this question of the individuality of the gut microbiome using a robust longitudinal dataset from wild meerkats and a sophisticated statistical approach not yet applied to microbiome studies. The authors leverage almost 1000 samples collected from 157 meerkats across two decades. They examine individuality using not only beta dissimilarity between individuals and microbial community stability within an individual, but also by estimating the intraclass correlation coefficient (ICC). Estimating the ICC measures the repeatability of the microbiome by determining what proportion of total phenotypic variation is explained by individual identity. ICC for linear mixed-effects models is most often calculated as the ratio of the variance of the random effect of interest (here, individual) to total variance ($\mathrm{ICC}=\sigma_{\mathrm{individual}}^{2}/\sigma_{\mathrm{total}}^{2}$ or $\mathrm{ICC}=\sigma_{\mathrm{individual}}^{2}/(\sigma_{\mathrm{individual}}^{2}+\sigma_{\mathrm{residual}}^{2}$)). Several R packages include functions for calculating ICC, including *ICC*, *irr* and *psych* for linear models, and *rptR*, *rptGam* and *performance* for more complex models. ICC, which has not been applied to microbiome studies until now, can be used to examine individuality for multiple microbial community phenotypes in addition to whole microbial community composition, such as alpha (or within-sample) diversity, bacterial load, or the abundance of specific taxa.

This study finds that individual meerkat identity explains a large proportion of microbiome variation, but only over short timescales. When examining samples collected over a 22-year period, sampling year accounts for the largest proportion of variation. Gut microbiome community composition, within-sample diversity (measured using both richness and evenness), and individual taxa abundances are not constant within an individual or social group over long periods of time. Instead, there are consistent effects of the environment on all individuals within the population—a year with high species richness for one individual is a year with high species richness for all individuals. Some aspects of gut microbial variation are more individualized, particularly those that are more closely tied to host genetics or that are known to be vertically transmitted. The abundances of highly heritable taxa show more repeatability within an individual than other taxa.

While the results here convincingly show that meerkat gut microbiomes are not individualized over longer periods of time, this may not be the case for all species. Host diet, physiology, ecology and phylogeny will all play a role in how individualized a species' microbiome is. Species with high microbial turnover rates due to diet, gut transit times or rapid changes in microbial composition over the course of a day (as is seen in this population of meerkats [10]) are less likely to have individualized microbiomes, as environmental microbe acquisition and transient microbial colonization would gain importance. For example, in humans, the similarity of the gut microbiome within an individual was lower when consuming an animal-based diet than when consuming a plant-based diet [11].

The authors not only use an innovative statistical approach to address the question of microbiome individuality, they also provide a statistical framework for exploring the strength and importance of host-related factors or traits (e.g. host physiology and genetics) in shaping microbiome composition (figure 1). By estimating the ICC for individual and time (as well as other host- or environment-related factors) for multiple measures of microbial community phenotypes, we can ask when host differences are more important than environmental factors in shaping host-associated microbial community composition. When do we see individualized microbial community dynamics, individualized microbial community composition, both or neither?

**Figure 1.**
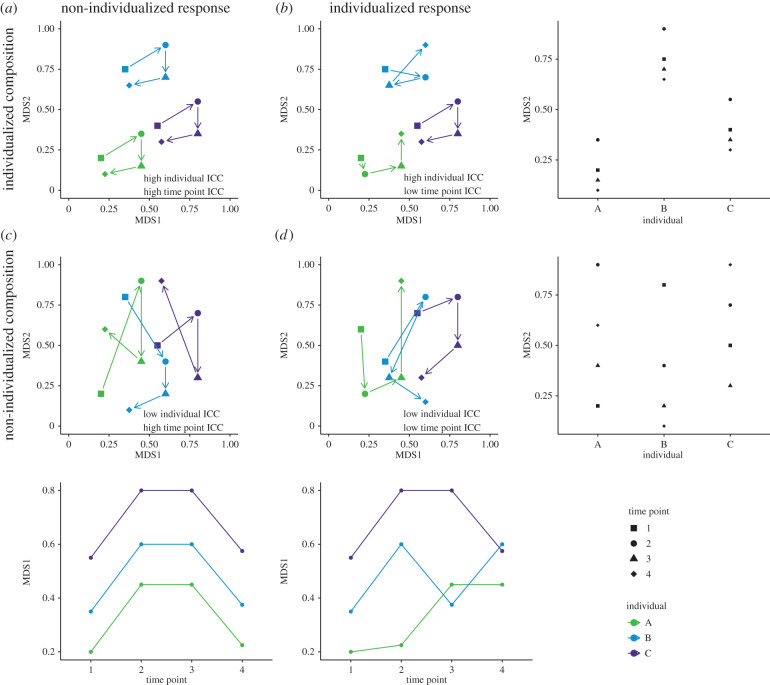
Microbial community composition over time under different hypothetical scenarios. Microbial community composition can be individualized (*a*,*b*) or not (*c*,*d*), and microbial community dynamics can be individualized (*b*,*d*) or not (*a*,*c*). The change in MDS1 over time is shown along the bottom, and the difference in MDS2 between individuals is shown along the right-hand side. (Online version in colour.)

In case A, when examining beta similarity between individuals, we could see individualized microbial community composition where individuals have a fairly static microbiome, but not an individualized response. Instead, every individual in the population responds similarly to an environmental change. In this case, both individual and time point will have a high ICC. In case B, microbial community composition is still individualized, but the response to environmental change is also individualized. In this case, individual but not time point will have a high ICC. In case C, microbial community composition is not individualized and there is substantial overlap between individuals over time. Additionally, community dynamics in response to environmental change are similar. In this case, time point but not individual will have a high ICC. Finally, in case D, microbial community composition is not individualized but microbial community dynamics are. In this case, neither individual nor time point will have a high ICC.

Relying on pairwise beta similarity approaches will detect individualized composition and distinguish between an individualized and non-individualized response in cases A and B. The magnitude of within-individual differences between time points will differ across individuals when the response is individualized. However, beta similarity approaches would not be able to distinguish between individualized and non-individualized responses in the absence of individualized composition. In cases C and D, the magnitude of within-individual differences between time points could differ across individuals for both an individualized response and a non-individualized response, depending on which microbial phenotype is examined.

Characterizing a host-associated microbial community as having individualized or non-individualized composition and individualized or non-individualized responses can provide clues as to the relative strengths of various eco-evolutionary processes that shape microbial community composition [12,13]. Microbiomes with individualized compositions are likely subject to strong host influences on microbial selection via host physiological filtering, strong priority effects (where early microbial colonizers outcompete microbes that are acquired later), high rates of vertical transmission and/or low rates of horizontal transmission. By contrast, non-individualized compositions may result from higher host immune tolerance or high rates of acquisition of microbes from the environment and conspecifics. Individualized community responses could occur when there are strong host influences on microbial community diversification where host genetic and physiological differences result in variation in microbial community dynamics. Interrogating multiple aspects of microbial community phenotypes by estimating the ICC of various factors can further disentangle the relative contributions of host and environmental factors. For example, the abundance of taxa that are environmentally acquired should be strongly associated with time and not individual identity, but the abundance of taxa that are vertically transmitted should be strongly associated with individual identity.

Risely *et al*. highlight that understanding the conditions that favour specific eco-evolutionary processes, such as high host genetic control and/or low horizontal transmission leading to more stable and/or individualized microbiomes, is important for understanding the selective pressures shaping the evolution of host–microbe associations. This work not only shows that meerkat gut microbiomes do not have an individualized composition over longer periods of time, the authors also illustrate the utility of applying novel statistical approaches to microbiome data to address key questions in the field. This framework can be used to ask why some mammals exhibit phylosymbiosis and others do not. For example, we might expect to see less individualized compositions or responses in clades that do not exhibit phylosymbiosis. In addition, this framework can be used to examine the contributions of the gut microbiome to host evolution by asking if highly individualized responses to environmental change influence fitness. It can also be used to determine if the contributions of environmental factors to microbiome dynamics are context dependent by investigating if the magnitude of seasonal environmental change shapes the individuality of composition within a species. Risely *et al.* have provided a roadmap for examining how environmental factors, host traits and microbial traits collectively shape host-associated microbial community dynamics.

## Data Availability

Data and code used to generate figure 1 can be found on GitHub (https://github.com/Mallott-Lab/ICC_commentary).

## References

[1] Ren T, Boutin S, Humphries MM, Dantzer B, Gorrell JC, Coltman DW, McAdam AG, Wu M. 2017 Seasonal, spatial, and maternal effects on gut microbiome in wild red squirrels. Microbiome **5**, 163. (10.1186/s40168-017-0382-3) 29268780 PMC5740981

[2] Sadoughi B, Schneider D, Daniel R, Schülke O, Ostner J. 2022 Aging gut microbiota of wild macaques are equally diverse, less stable, but progressively personalized. Microbiome 10, 95. (10.1186/s40168-022-01283-2) 35718778 PMC9206754

[3] Turnbaugh PJ et al. 2009 A core gut microbiome in obese and lean twins. Nature **457**, 480-484. (10.1038/nature07540) 19043404 PMC2677729

[4] Degnan PH, Pusey AE, Lonsdorf EV, Goodall J, Wroblewski EE, Wilson ML, Rudicell RS, Hahn BH, Ochman H. 2012 Factors associated with the diversification of the gut microbial communities within chimpanzees from Gombe Naitonal Park. Proc. Natl Acad. Sci. USA **109**, 13 034-13 039. (10.1073/pnas.1110994109) PMC3420156 22826227

[5] Orkin JD, Campos FA, Guadamuz A, Melin AD, Myers MS, Hernandez SEC. 2019 Seasonality of the gut microbiota of free-ranging white-faced capuchins in a tropical dry forest. ISME J. **13**, 183-196. (10.1038/s41396-018-0256-0) 30135468 PMC6298967

[6] Ren T, Grieneisen LE, Alberts SC, Archie EA, Wu M. 2016 Development, diet and dynamism: longitudinal and cross-sectional predictors of gut microbial communities in wild baboons. Environ. Microbiol. **18**, 1312-1325. (10.1111/1462-2920.12852) 25818066 PMC5941927

[7] Amato KR et al. 2015 The gut microbiota appears to compensate for seasonal diet variation in the wild black howler monkey (*Alouatta pigra*). Microb. Ecol. **69**, 434-443. (10.1007/s00248-014-0554-7) 25524570

[8] Garud NR, Good BH, Hallatschek O, Pollard KS. 2019 Evolutionary dynamics of bacteria in the gut microbiome within and across hosts. PLoS Biol. **17**, e3000102. (10.1371/journal.pbio.3000102) 30673701 PMC6361464

[9] Risely A, Schmid DW, Müller-Klein N, Wilhelm K, Clutton-Brock TH, Manser MB, Sommer S. 2022 Gut microbiota individuality is contingent on temporal scale and age in wild meerkats. Proc. R. Soc. B **289**, 20220609. (10.1098/rspb.2022.0609) PMC9382201 35975437

[10] Risely A, Wilhelm K, Clutton-Brock T, Manser MB, Sommer S. 2021 Diurnal oscillations in gut bacterial load and composition eclipse seasonal and lifetime dynamics in wild meerkats. Nat. Commun. **12**, 6017. (10.1038/s41467-021-26298-5) 34650048 PMC8516918

[11] David LA et al. 2014 Diet rapidly and reproducibly alters the human gut microbiome. Nature **505**, 559-563. (10.1038/nature12820) 24336217 PMC3957428

[12] Kohl KD. 2020 Ecological and evolutionary mechanisms underlying patterns of phylosymbiosis in host-associated microbial communities. Phil. Trans. R. Soc. B **375**, 20190251. (10.1098/rstb.2019.0251) 32200746 PMC7133527

[13] Mallott EK, Amato KR. 2021 Host specificity of the gut microbiome. Nat. Rev. Microbiol. **19**, 639-653. (10.1038/s41579-021-00562-3) 34045709

