# Correction to: ‘Individualized composition or community dynamics? A new statistical approach to assess the individuality of host-associated microbiomes’ (2022) by Mallott

**DOI:** 10.1098/rspb.2022.2333

**Published:** 2022-12-21

**Authors:** Elizabeth K. Mallott

*Proc. R. Soc. B* **289**, 20221794. (Published online 9 November 2022) (https://doi.org/10.1098/rspb.2022.1794)

The original article contains an incorrect citation to Touitou *et al.* 2021 ([2] in the original article).

The second reference should instead read:

Sadoughi B, Schneider D, Daniel R, Schülke O, Ostner J. 2022 Aging gut microbiota of wild macaques are equally diverse, less stable, but progressively personalized. *Microbiome* **10**, 95. (doi:10.1186/s40168-022-01283-2)

